# PAX4 Enhances Beta-Cell Differentiation of Human Embryonic Stem Cells

**DOI:** 10.1371/journal.pone.0001783

**Published:** 2008-03-12

**Authors:** Chee Gee Liew, Nadia N. Shah, Sarah J. Briston, Ruth M. Shepherd, Cheen Peen Khoo, Mark J. Dunne, Harry D. Moore, Karen E. Cosgrove, Peter W. Andrews

**Affiliations:** 1 Centre for Stem Cell Biology, Department of Biomedical Science, The University of Sheffield, Sheffield, United Kingdom; 2 Centre for Stem Cell Biology, Department of Molecular Biology and Biotechnology, The University of Sheffield, Sheffield, United Kingdom; 3 Faculty of Life Sciences, University of Manchester, Manchester, United Kingdom; Baylor College of Medicine, United States of America

## Abstract

**Background:**

Human embryonic stem cells (HESC) readily differentiate into an apparently haphazard array of cell types, corresponding to all three germ layers, when their culture conditions are altered, for example by growth in suspension as aggregates known as embryoid bodies (EBs). However, this diversity of differentiation means that the efficiency of producing any one particular cell type is inevitably low. Although pancreatic differentiation has been reported from HESC, practicable applications for the use of β-cells derived from HESC to treat diabetes will only be possible once techniques are developed to promote efficient differentiation along the pancreatic lineages.

**Methods and Findings:**

Here, we have tested whether the transcription factor, Pax4 can be used to drive the differentiation of HESC to a β-cell fate *in vitro*. We constitutively over-expressed Pax4 in HESCs by stable transfection, and used Q-PCR analysis, immunocytochemistry, ELISA, Ca^2+^ microfluorimetry and cell imaging to assess the role of Pax4 in the differentiation and intracellular Ca^2+^ homeostasis of β-cells developing in embryoid bodies produced from such HESC. Cells expressing key β-cell markers were isolated by fluorescence-activated cell sorting after staining for high zinc content using the vital dye, Newport Green.

**Conclusion:**

Constitutive expression of Pax4 in HESC substantially enhances their propensity to form putative β-cells. Our findings provide a novel foundation to study the mechanism of pancreatic β-cells differentiation during early human development and to help evaluate strategies for the generation of purified β-cells for future clinical applications.

## Introduction

Pancreatic β-cells are the primary source of physiologically-relevant insulin and defects in their function cause diabetes and hyperinsulinism. Several groups have reported evidence for the presence of cells resembling β-cells among the differentiated derivatives formed in EBs of HESC [1], [2]. Others have also found enhanced differentiation of such cells from HESC and mouse embryonic stem cells (MESC) after culturing EBs in media that selectively promote the growth of neuroectodermal cells [3], [4], [5], [6]. As some of these techniques have proved unreliable and difficult to replicate [7], [8], attention has switched to testing whether specific signalling pathways that guide the appearance of β-cells during embryonic development can be applied to MESC or HESC *in vitro*.

During embryonic development, the pancreatic primordium arises from the posterior foregut region of the definitive endoderm, in a step that is dependent upon the transcription factor Pdx1. Thus, homozygous knock-out mice lacking Pdx1 develop the pancreatic buds but fail to form a pancreas [9], [10]. Subsequently, Pdx1 expression down-regulates and fate restriction of various cells in the pancreatic primordium results in the formation of distinct exocrine and endocrine cells [11]. Building on these observations, D'Amour et al [12] found that after inducing definitive endoderm differentiation from HESC, the subsequent exposure to retinoic acid and an inhibitor of hedgehog signalling could lead to the formation of cells expressing insulin. On the other hand, although Lavon et al [13] found that over-expression of Pdx1 in HESC enhanced pancreatic endocrine cell differentiation in EBs, they failed to find evidence of β-cell formation *in vitro.* We speculated that signals induced downstream of definitive endoderm might be, at least in part, more potent to trigger subsequent signal cascades that culminate with the pancreatic β-cell formation.

The precise developmental relationship of the cell lineages in the human pancreas remains uncertain, but in mice the generation of β-cells is specifically dependent upon the transcription factor Pax4. Inactivation of Pax4 by homologous recombination resulted in the absence of mature insulin-producing β-cells in the pancreas of Pax4 homozygous mutant mice [14]. This suggests a role for Pax4 in committing early pancreatic endocrine cells to a β-cell fate, although it has also been demonstrated that Pax4 expression can generate other islet endocrine cells [15]. Based on its onset of activation prior to β-cell specification in developing pancreas, Blyszczuk et al. (2003) showed that over-expression of Pax4 in MESC enhanced the expression of β-cell genes and insulin [16]. However, since significant differences have been documented between the behaviour of MESC and HESC [17], and as several studies also revealed differences between mouse and human embryogenesis [18], [19], we sought to determine whether Pax4 expression can be harnessed *in vitro* to enhance differentiation of HESC into β-cells.

## Materials and Methods

### Cell Culture and Transfection

A subline of H7 HESC (WiCell Research Institute, Madison, WI), H7.S6 was used throughout the study. This subline was adapted to culture, which allowed efficient passaging and cloning to facilitate transfection, while retaining the capacity for extensive differentiation [20]. Briefly, cells were cultured in HESC medium (knockout-DMEM supplemented with 20% Serum Replacement, 1% non-essential amino acids, 1 mM L-glutamine, 0.1 mM β-mercaptoethanol [Sigma-Aldrich, Poole, UK] and 4 ng/ml basic FGF) under a humidified atmosphere of 5% CO_2_ in air at 37°C. For sub-cultivation, the cells were harvested by treatment with 1 mg/ml collagenase type IV in DMEM:F12 per T25 flask for 8 to 10 minutes at 37°C, dispersed by scraping with 3mm glass bead, centrifuged at 68×g for 3 minutes and then seeded onto inactivated mouse embryonic fibroblast (MEF) feeders that had been washed once with phosphate-buffered saline (PBS) immediately prior to use.

The construct used to generate pCAG-PAX4 expression vector was made with pCAGeGFP vector [21]-by replacing eGFP with the human *Pax4* gene coding sequence (CDS) (located at 207-1238 base pairs (bp) on *Homo sapiens Pax4* mRNA, Gene Bank Accession Number NM_006193) amplified from H7 EB cDNA. The *Pax4*-CDS PCR product was purified from 1% agarose gel with Qiagen gel purification kit (Qiagen, Crawley, UK), subcloned into pGEM-T easy vector (Promega, Southampton, UK) and released by *Not* I and *Xho* I restriction digestions. To remove eGFP, the parental pCAGeGFP vector was linearised by *Not* I and partially digested with *Sal* I. *Pax4* CDS was ligated into the 6.44kb fragment of pCAG vector with T4 ligase (Promega), generating pCAGPax4 vector (See Supplementary Information Fig. S1 for vector map).

H7 HESC were transfected using ExGen500 transfection reagent (MBI Fermentas, Germany) as previously described [21]. Briefly, cells were seeded one day prior to transfection with the initial seeding density of 3×10^5^ cells in a single well on 6-well plates. 0.05% trypsin/EDTA was used to harvest HESC; the cells were then seeded on matrigel-coated 6 well-plates and in MEF-conditioned medium prior to transfection (Matrigel from BD Biosciences, Oxford, UK). Cells were approximately 70% confluent on the day of transfection. Transfection was carried out with 9.5 µg plasmid DNA using ExGen500. For derivation of stable clones, transfected cells were subjected to antibiotic selection with 1 µg/ml puromycin (Sigma) 24 hours after transfection. Distinct, puromycin-resistant, individual colonies appeared after 2–3 weeks and were hand-picked by micropipette, dissociated into small clumps of cells, and transferred into one well of a 12-well culture dish. The cells (H7.Px4) were then expanded in 6-well plate and subsequently passaged into 25 cm^2^ tissue culture dishes.

### Embryoid Body Differentiation

*In vitro* differentiation of H7 and H7.Px4 cells was induced by aggregation of HESC in suspension culture. Briefly, undifferentiated HESC were harvested with 1 mg/ml collagenase IV as above, pelleted by centrifugation and resuspended in HESC medium containing all supplements as described above, and transferred to sterile 10 cm bacteriological Petri dishes (Sterilin, UK). The cells were incubated at 37°C in 5% CO_2_. The medium was replaced every other day and the resulting EBs were differentiated for different time points and collected for subsequent analysis.

### RT-PCR

Total RNA was isolated from HESCs and EBs using RNeasy mini kits (Qiagen, Crawley, UK). Samples were DNase treated (Ambion, Huntingdon, UK) and quantified using a NanoDrop spectrophotometer (NanoDrop, Labtech International, Ringmer, UK). RNA integrity was verified using an Agilent 2100 Bioanalyser (Agilent Technologies, Wokingham, UK). cDNA was synthesized from 1 µg RNA with Superscipt II reverse transcriptase and oligo (dT)_12–18_ primers. PCR was performed with gene-specific primers (500 nM) designed in-house (See Supplementary Information, Table S1), with Taq polymerase and associated reagents. For all reactions, controls included no-template, RT-positive and RT-negative samples to detect any gDNA contamination. PCR products were identified using standard ethidium bromide agarose gel electrophoresis and their identity was confirmed by sequencing gene products. All electronic gel images were cropped to show the single product band, related controls and molecular weight markers, and no further image manipulation was used.

### Real-time Quantitative PCR (Q-PCR)

Total RNA was isolated from HESCs and EBs as described above. cDNA was synthesized from 1 µg RNA with Superscript II reverse transcriptase and random hexamers or a 1∶3 mixture of random hexamers and oligo (dT)_12–18_ primers (Gibco, Paisley, UK). PCR was performed with gene-specific primers (150 or 300 nM) with Platinum SYBR Green qPCR Supermix , Power SYBR Green PCR Mastermix (Applied Biosystems, Warrington, UK) or Assay-on-Demand technology (Applied Biosystems, Warrington, UK). Human brain RNA (Clontech, Saint-Germain-en-Laye, France) was converted to cDNA and used to generate standard curves for subsequent voltage-gated Ca^2+^ channel gene expression studies. Q-PCR was performed in an ABI 7500 thermal cycler (Applied Biosystems, Warrington, UK) or iCycler iQ (Bio-Rad Laboratories Ltd., Hemel Hempstead, UK). A dissociation step was performed at the end of every experiment to confirm the presence of a single PCR product. ABI 7500 software and Excel spreadsheets (Microsoft UK, Reading, UK) were used to analyse the data. The baseline was manually set to 2 cycles below the start of any amplification and the threshold manually adjusted by choosing the value that gave the most precision between replicate samples. For the voltage-gated Ca^2+^ channel gene expression studies, samples were normalized to total RNA input with PCR product concentration determined from the standard curve and expressed relative to the calibrator sample (day 7 untransfected H7 EBs). For other gene expression studies, samples were normalized to 18sRNA and 2^ΔΔCt^ analysis applied. Data were calibrated relative to the day 7 untransfected H7 EBs as before. For comparison of transcript levels in unsorted and FACS-sorted cells, the difference in cycle times, ΔC_t_, was determined as the difference between the tested gene and the reference housekeeping gene, *ACTB*. PCR reactions for each sample were repeated in triplicates.

### Western Blotting

Cells were harvested into 50 µl 1× lysis buffer (50 mM Tris, pH 8.0, 150 mM NaCl, 1% (w/v) Triton X-100 (Sigma), 0.1% (w/v) SDS supplemented with a cocktail of protease inihibitor (Roche, UK). Each lane was loaded with 20 µg of total protein. Gels were blotted onto a nitrocellulose membrane (Bio-Rad) and incubated with an antibodies against human PAX4 (1∶500; Aviva, San Diego, CA) or an anti-β actin antibody (1∶1,000; Abcam) overnight at 4°C, followed by incubation for 1-h with secondary anti-rabbit antibody conjugated to HRP (Santa Cruz, Autogen-Bioclear, Calne, UK). Membranes were developed using ECL Western blotting detection system (Amersham Biosciences, Cardiff, UK) according to manufacturer's protocol.

### Immunocytochemistry

Cells were washed twice with PBS and fixed in 4% paraformaldehyde in PBS for 20 min. Cells were then washed three times in PBS and incubated for 10 min in PBS with 0.1% Triton-X. After blocking with PBS with 0.1% Triton-X and 1% sheep serum (blocking solution) for 30 min, cells were incubated with primary antibodies-either anti-PAX4 (1∶200; Aviva, San Diego, CA), anti-Proinsulin (1∶100; Autogen Bioclear), MC631 (anti-SSEA3, 1∶100), TRA-1-60 (1∶100) for overnight at 4°C. MC631 and TRA-1-60 antibodies were prepared as supernatants of the hybridomas grown in our laboratory as previously described [22], [23]. Cells were then washed in blocking solution and incubated with Cy3 goat anti-mouse IgG (1∶300; Sigma, Dorset, UK) or FITC-conjugated goat anti-rabbit IgG (1∶200; Stratech, Newmarket, UK) for one hour at room temperature, followed by three washes with blocking solution. Immunofluorescence with the specific antibodies was compared with that from negative control antibodies obtained from parent myeloma cell line P3×63Ag8 [22] and rabbit IgG FITC secondary antibody (1∶100; Abcam, Cambridge, MA, USA) to indicate specificity. Cells were mounted with DAPI mounting reagent (Vectashield, Vector Labs, Peterborough, UK) that will also counterstain nuclei. Images were captured with an Olympus CKX41 microscope and Nikon Coolscope DS-5M digital camera at 5MP resolution.

### Fluorescence Labeling with Newport Green

16–21 day old EBs were allowed to attach on Matrigel and were further differentiated for 7–10 days as monolayer in 1∶1 DMEM (no glucose)-F12 medium supplemented with 1% FCS and 10 mM nicotinamide (Sigma). Newport Green diacetate, NG-Ac (Molecular Probes Europe, Leiden, The Netherlands) was used for fluorescent studies on living cells. Cells were washed twice with PBS and then incubated for 3 min at 37°C with PBS containing 1 µM NG-Ac containing 1 µl/ml Pluronic F127 (Molecular Probes) to aid penetration of the probe. After washing in PBS with 5% FCS, the cells were dissociated and the single cell suspension was subjected to confocal microscopy (Nikon Eclipse TE300) and FACS analysis.

### FACS Analysis and Sorting

Cells were harvested with 0.25% trypsin/EDTA and resuspended at 2×10^6^ cells per ml in wash buffer (5% FCS) and 0.1% sodium azide (Sigma) in PBS and analyzed on a MoFlow FACS sorter (Dako Cytomation, Ely, UK). NG-labeled cells were analyzed by FACS according their fluorescence emission (excitation 488 nm). The negative control was undifferentiated cells and was used to set the background level of fluorescence. Cell samples were sorted for the collection of 2×10^4^–1×10^6^ cells in 10 ml conical tubes. Following sorting, cells were either collected for RNA analysis, or were further grown in 1:1 DMEM (no glucose)-F12 medium containing 1% FCS and 10 mM nicotinamide. Sorted cells were replated at the density of 3×10^3^ cells per cm^2^ on 0.1 mg/ml human placental collagen IV-coated tissue culture dishes.

### C-Peptide Content and Secretion

Following the designated differentiation periods of 7, 14 and 21 days, 100 EBs were selected for measurement of C-peptide content at each time point. EBs of a regular size and morphology were transferred to a glass tube, washed in Krebs Ringer buffer supplemented with 1 mg/ml bovine serum albumin (Insulin RIA grade, Sigma) and cell lysis performed as described previously [24]. Samples were stored at −20°C prior to analysis. C-peptide content was assessed using a Human C-peptide ELISA kit according to the manufacturer's Instructions (Mercodia Ultrasensitive, Diagenics, Milton-Keynes, UK). The effect of tolbutamide and glucose on C-peptide release was studied in parallel with NG-positive or NG-negative cells. An initial one hour incubation was carried out in Krebs Ringer buffer containing 2.2 mM D-glucose (Roche), and then with 100 µM tolbutamide (Sigma) or 27 mM glucose for 15 min. The fold stimulation was calculated for each condition by dividing the C-peptide concentration released in response to stimulatory conditions by the C-peptide concentration in the basal medium.

### Functional Changes in Cytosolic Ca^2+^ Signaling

Prior to experimentation, EBs were resuspended in hES medium supplemented with penicillin/streptomycin (100 U/0.1 mg). Following overnight culture on poly-D-ornithine-coated coverslips (Sigma, 10 µg/ml) in 24-well plates, the cytosolic Ca^2+^ concentration ([Ca^2+^]_i_) was measured by digital imaging microfluorimetry (Roper Scientific, Marlow, Bucks, UK). The EBs were loaded with Fura 2-AM to a final concentration of 6 µM for up to 90 minutes at 37°C where the coverslip formed the base of a perifusion chamber (Warner Instruments, Harvard Apparatus, Edenbridge, Kent, UK). Excitation of the sample EBs at 340 and 380nm was achieved by a monochromator (T.I.L.L. Photonics, Planegg, Germany) with a cycle time of 1.32 seconds. Image capture was performed by a Quantix photometrics CCD camera (Roper Scientific). During experimental procedures the control solution consisted in mM: NaCl 137, KCl 5.36, MgSO_4_ 0.81, Na_2_HPO_4_ 0.34, KH_2_PO_4_ 0.44, CaCl_2_ 1.26, NaHCO_3_ 4.17, HEPES 10 and glucose 2.02. The pH was set to 7.4 using NaOH. In solutions containing a high concentration of K^+^, NaCl was replaced by equimolar KCl. An *in vitro* calibration procedure was performed to determine an estimation of changes in [Ca^2+^]_i_ [25]. For the purposes of quantification, the functional capacity of EBs has been defined as the product of the percentage of responding EBs and the average change in [Ca^2+^]_i _under a specific condition. These values have been normalized to the early time-point control. Ca^2+^ responses were calculated as the peak rise in [Ca^2+^]_i_ from its level immediately prior to stimulation. The average percentage response for each EB was determined by combining the sizes of the areas which showed a [Ca^2+^]_i_ rise of over 10 nM and expressing them as a percentage of the area of the entire EB. All measurements are recorded as means±SEM.

### Chemicals and Reagents

Chemicals were from Invitrogen, Paisley, UK unless otherwise stated. For imaging studies, stocks of ATP were made as 100 mM in distilled H_2_O and were maintained at −20°C. Tolbutamide was made as 100 mM in DMSO and used 1∶1,000 in Krebs Ringer buffer for secretion experiments.

### Statistical analysis

All averaged data are expressed±standard error of the mean unless otherwise stated. Statistical analysis was performed on the data using SigmaStat (Systat, Hounslow, UK). For comparisons of more than 3 pairs of data, one-way analysis of variance was performed prior to pairwise comparisons using Bonferonni's test or Holm-Sidak test. For comparisons of discrete data sets, unpaired Student's t-tests were used. Significance levels or p values are stated in figure legends.

## Results

### Stable Transfection of HESC with Pax4

**Figure 1:**
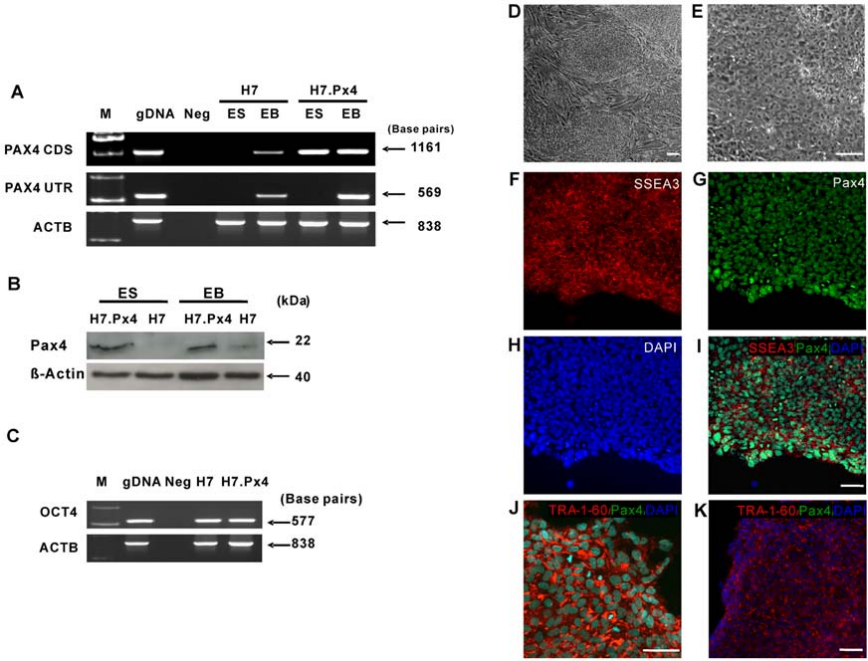
Human embryonic stem cells (HESC) stably transfected with human *Pax4* coding sequence. (A) Expression levels of *Pax4* gene during HESC differentiation measured by semi-quantitative RT-PCR in undifferentiated embryonic stem cells (ES) and embryoid bodies (EBs) of untransfected H7 and H7.Px4 cells stably transfected with human *Pax4* coding sequence (CDS). The RT-PCR for the untranslated region of *Pax4* (PAX4 UTR) only detects endogenous expression whereas the RT-PCR for CDS picks up both endogenous and transgene expression. Genomic DNA (gDNA) was used as positive control and PCR without cDNA was used as negative control (Neg). *ACTB* (β-actin) was used as loading control in all samples. All five clones of H7.Px4 cells behaved similarly. (B) Western blot analysis in undifferentiated and late stage differentiated (21 days) EBs of H7 and Pax4-expressing H7 cells. β-actin was used as loading control in all samples. (C) Undifferentiated H7.Px4 cells expressed high level of *Oct4* indistinguishable from that in untransfected H7 cells. (D, E) Undifferentiated H7.Px4 cells also displayed a morphology characteristic of undifferentiated HESC. (F–K) As shown by immunocytochemistry, H7.Px4 HESC express human Pax4 protein (*green*), stage specific embryonic antigen-4 (SSEA4) (*red*) and TRA-1-60 (J). Nuclei were stained with DAPI (*blue*). Note the turquoise colour of the nuclei resulting from the overlay of Pax4 and DAPI staining. Scale bars, 50 µm. Human Pax4 protein is not expressed by untransfected H7 HESC (K). All data are typical of at least 2 experiments on control and from at least 3 independent H7.Px4 clones.

We first examined the expression of Pax4 in HESC and their differentiated derivatives. Neither mRNA nor protein were detected for Pax4 in undifferentiated H7 HESC, by RT-PCR or Western blotting respectively. To induce *in vitro* differentiation of HESC, we applied the suspension method used for EB formation, which resulted in an increased, but low level expression of Pax4 mRNA and protein (Figure 1A and 1B). We isolated the coding sequence (CDS) for human *Pax4* by PCR from differentiated H7 EBs and inserted it into the pCAG vector upstream of an IRES linking it to the puromycin resistance gene (see Supplementary Information, Figure S1). Previously, we found that this vector can be used to derive stable transfectants of HESC without subsequent gene silencing [21]. Following transfection and selection with puromycin we isolated several independent clones of H7 HESC, three of which were used in subsequent studies (H7.Px4).

All the undifferentiated H7.Px4 transfectants expressed the *Pax4* transgene, detected by RT-PCR for the CDS region, but not the endogenous gene detected by RT-PCR for the 5′UTR, which was absent from the transgene (Figure 1A). During differentiation, the expression of the endogenous *Pax4* mRNA was substantially increased in the H7.Px4 EBs, and its induction occurred earlier than in the EBs of untransfected cells (Figure 1A; data not shown). Most likely, this reflects a positive transcriptional feedback loop since the *Pax4* promoter contains several binding sites for Pax4 itself [26]. Pax4 protein was also strongly expressed in the transfected cells compared to the untransfected cells (Figure 1B, 1G, 1K). Before induction of differentiation, the transfected cells retained an undifferentiated phenotype despite their expression of Pax4. They expressed *Oct4* similarly to the untransfected cells (Figure 1C) and retained the morphology (Figure 1D and 1E) and surface antigen expression markers (e.g. stage specific embryonic antigen-3 (SSEA3) and TRA-1-60) of undifferentiated HESC [27] (Figure 1F–1J).

### Upregulation of β-cell Transcripts in H7.Px4 EBs during Differentiation

**Figure 2:**
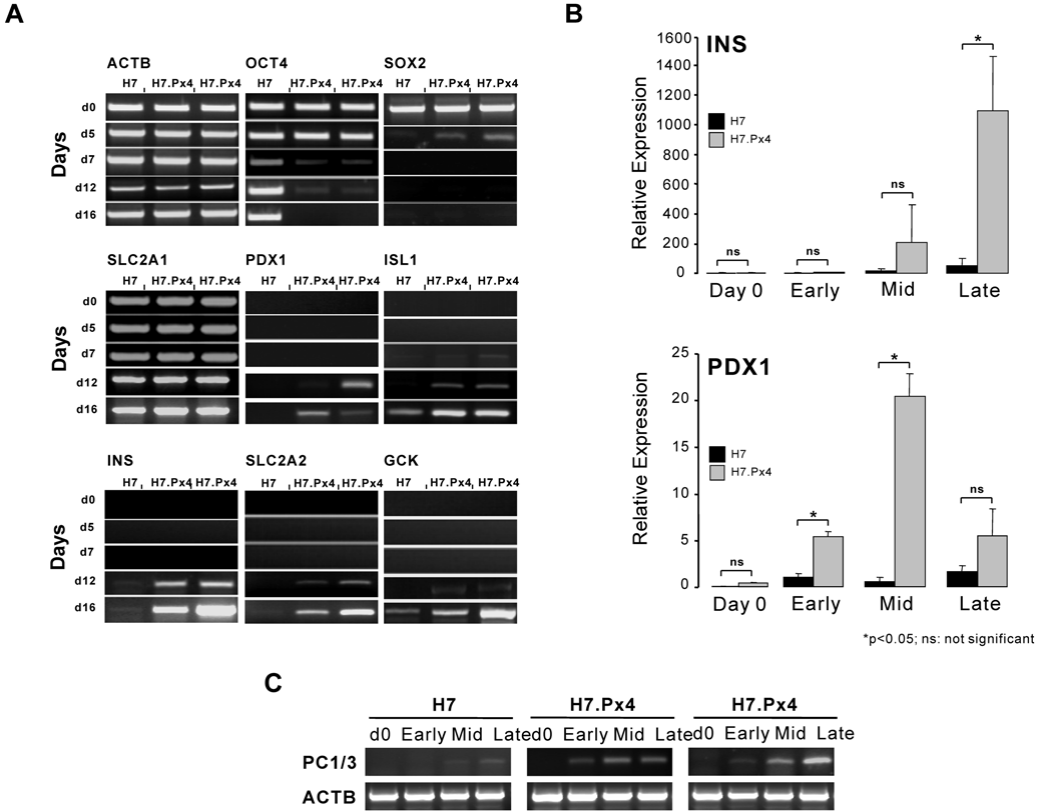
The effect of Pax4 expression upon EB differentiation of HESC. (A) RT-PCR analysis of gene expression in EBs produced from untransfected H7 cells and from two independent clones transfected with *Pax4* during a 16 day in vitro differentiation. (B) Q-PCR analysis of *Ins* and *Pdx1* expression in EBs produced from untransfected H7 and H7.Px4 cells. These experiments were performed on two independent H7.Px4 clones and control data came from 3 independent experiments using untransfected H7 cells. Data were collected on Day 0 and at 7 (Early), 14 (Mid) and 21 (Late) days following EB formation. Gene expression levels were calculated as 2^−ΔΔCt^ values, relative to day 7 (Early) samples. Error bars represent standard deviation, and an asterisk denotes p<0.05. (C) Differentiation of H7.Px4 EBs was also associated with the up-regulated expression of gene encoding enzyme prohormone convertase 1/3 (PC1/3).

To determine the behaviour of H7.Px4 cells during their growth as EBs, we next examined the expression of a panel of cell-specific genes and proteins during EB differentiation (Figure 2A). In untransfected and H7.Px4 cells, the expression of *Oct4* was down-regulated during EB growth over a 16 day period, though significant expression of *Oct4* was retained, and indeed increased at later time points in the untransfected cells. This retention of *Oct4* in EBs has been noted in other studies [28] and may reflect the persistence of undifferentiated cells and/or the appearance of cells that also express *Oct4*, such as those of the germ line [29]. In the H7.Px4 cells, there was a greater down-regulation of *Oct4*, and delay in disappearance of *Sox2*, which is expressed by cells of the neural lineage [30]. In addition, we observed strong expression of the endodermal transcription factor forkhead box A2 (*Foxa2*) in the EBs, indicating HESC could efficiently contribute to definitive endoderm germ layer during spontaneous differentiation [12], Supplementary Information, Figure S2.

The differential expression of β-cell and neural-specific transcription factors makes it possible to distinguish HESC-derived β-like cells from neural derivatives. No significant differences were observed between H7 and H7.Px4 EBs for expression of *SLC2A1*, which encodes a constitutive glucose transporter expressed in most human tissue types [31], or the K_ATP_ channel genes, *KCNJ11* and *ABCC8* (Supplementary Information, Figure S2). However, there were marked differences in the expression of several genes associated with the β-cell lineage [32], [33]. *Pdx1*, *Islet-1* (*Isl1*), *Ins*, *SLC2A2*, *Gck* and *PC1/3* (Figure 2A, 2C) were all expressed more strongly, with an earlier onset in the EBs from the H7.Px4 cells compared to the EBs from the untransfected cells. By itself, induction of *Ins* expression is suggestive but limited as a marker of ‘true’ β-cell differentiation [19]. However, the expression of additional markers characteristic of functionally competent cells such as *SLC2A2*, *Gck*, *Pdx1* and *PC1/3* [19], [34], provides further support for the appearance of β-cells in H7.Px4 EBs. To confirm these results we also used quantitative PCR (Q-PCR) to examine the expression of *Ins* and *Pdx1* in EBs from H7.Px4 clones (Figure 2B). In each case, the EBs from the H7.Px4 clones showed enhanced expression of both *Ins* and *Pdx1* compared to the untransfected cells, consistent with the expression of Pax4 promoting differentiation towards the β-cell lineage. Similar results were also obtained for *Gck*, *Isl1* and *SLC2A2* (data not shown). We also noted a significant influence of Pax4 expression on somatostatin and glucagon gene transcripts (Supplementary Information, Figure S2, S3). Taken together, these gene expression patterns are consistent with enhanced pancreatic endocrine and β-cell differentiation in the EBs from H7.Px4 cells.

### Physiological Assessment of Voltage-gated Ca^2+^Channels in EBs

**Figure 3:**
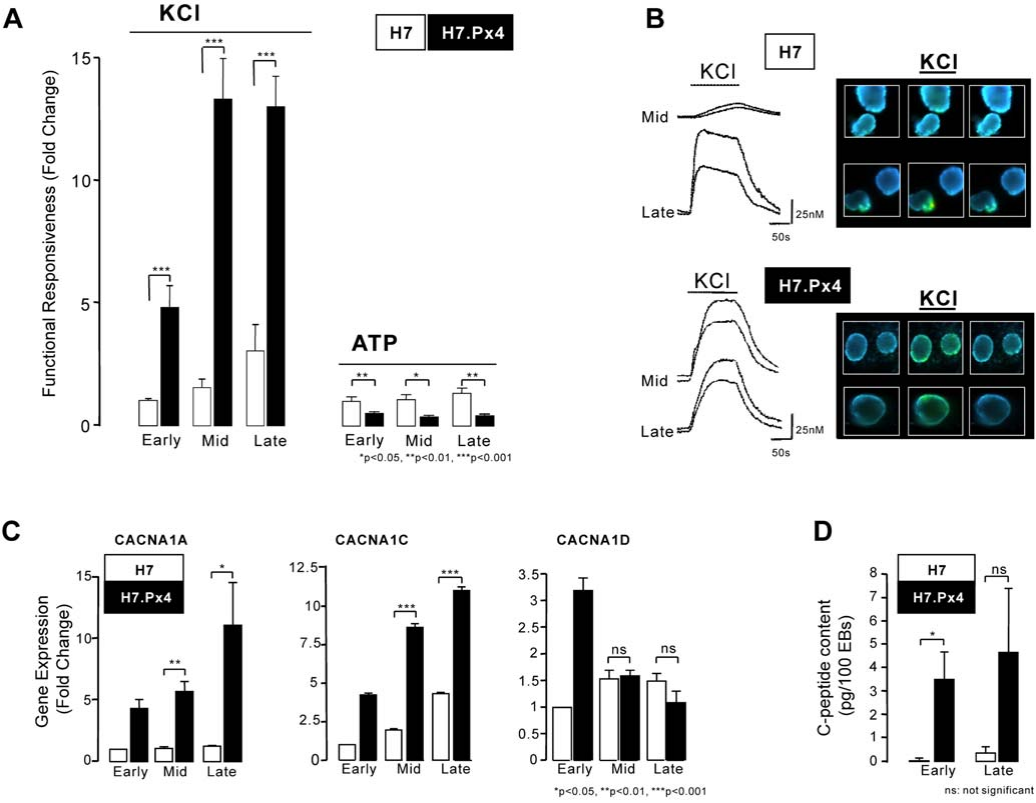
The expression and function of voltage-gated Ca^2+^ channels in H7 cells transfected with *Pax4.* (A) Selective enhancement and induction of functional responses in H7.Px4 EBs (n = 27–34 EBs) compared to untransfected H7 cells (n = 31–50 EBs). Note that the actions of ATP are associated with a declining functional capacity in H7.Px4 (n = 18–29 EBs), but not untransfected H7 cells (n = 44–52 EBs). (B) Typical VGCC-mediated responses in [Ca^2+^]_i _in H7 and H7.Px4 cells, respectively. (C) A summary of *CACNA1A*, *CACNA1C* and *CACNA1D* gene expression analyzed by Q-PCR in H7 and H7.Px4 cells. Data were obtained from n = 3 experiments on H7 controls and n = 3 experiments on H7.Px4 EBs from 1 H7.Px4 clone; similar trends were observed in another H7.Px4 clone (data not illustrated). (D) Production of C-peptide in early and late stage differentiating EBs from H7 and H7.Px4 cells. With the exception of Panel (C) all data were obtained from 3 independent experiments using untransfected H7 EBs and 3 independent H7.Px4 clones. Asterisk indicates p<0.05, double asterisk indicates p<0.01 and triple asterisk indicates p<0.001.

To explore further the nature of the cells induced in the EBs from the *Pax4*-transfected cells, we examined the dynamics of intracellular Ca^2+^ signals of H7 and H7.Px4 EBs to depolarization of the cell membrane and voltage-gated Ca^2+^ channel (VGCC) gene expression (Figure 3). Pancreatic β-cells are electrically active and regulate Ca^2+^ in response to a membrane depolarization via VGCCs. While glucose results in depolarization and subsequent Ca^2+^ influx into β-cells, various secretagogues, such as potassium choride (KCl), ATP and tolbutamide also trigger a similar response in the absence of glucose. We took advantage of a fluorescent indicator molecule Fura-2 AM, that enabled us to study the changes in intracellular free Ca^2+^ concentration ([Ca^2+^]_i_) using dual-wavelength excitation microfluorimetry in Fura-2-loaded EBs. In untransfected H7 cells, most EBs–71% to 78%, failed to respond to depolarizing concentrations of KCl (40 mM) between 7 and 21 days (at 7–9 days of differentiation positive responses were seen in 11/50 EBs, 11/44 EBs responded at 14–16 days and 9/31 EBs at 21–23 days). Over the same time points the average rises in the [Ca^2+^]_i _were 16±2 nM (n = 11) at the early time point, 20±5 nM (n = 11) in the mid-stage of EB differentiation and 39±15 nM (n = 9) in the oldest EBs. The functional responsiveness of the undirected differentiation pathway in H7 cells was therefore associated with a 3-fold increase in VGCC-dependent Ca^2+^ signalling (Figure 3A). In comparison, EBs from H7.Px4 cells were already approximately 5-fold more responsive at day 7 when compared to controls (48% of EBs responding with an average increase [Ca^2+^]_i _of 36±4 nM) and 13 fold more responsive at day 14 (30/30 EBs responded with an average increase [Ca^2+^]_i _of 48±6 nM) and day 21 (34/34 EBs responded with an average increase [Ca^2+^]_i _of 47±4 nM) (Figure 3A and 3B).

Whilst Pax4 led to the induction of marked increases in voltage-gated Ca^2+^ channel operation, ATP-induced changes in [Ca^2+^]_i_ which are mediated via purinergic receptors, independently of a change in the cell membrane potential, were decreased at all time-points in H7.Px4 cells. To understand the induction of voltage-gated Ca^2+^ signals we examined the expression of VGCC genes associated with the endocrine pancreas [35]. By RT-PCR we found *CACNA1S* (Ca_V_1.1), *CACNA1C* (Ca_V_1.2), *CACNA1D* (Ca_V_1.3), *CACNA1A* (Ca_V_2.1), *CACNA1B* (Ca_V_2.2) and *CACNA1E* (Ca_V_2.3), and *CACNB1* to *4*, (Ca_V_β1 to 4) mRNAs were all expressed in EBs from H7 and H7.Px4, and also in the human fetal pancreas (data not shown). Q-PCR was used to examine *CACNA1C*, *CACNA1D* and *CACNA1A* over time in H7 and H7.Px4 (Figure 3C). Pax4 was found to potentiate the expression of *CACNA1A*, induce a transient increase in *CACNA1D* and enhanced *CACNA1C* mRNA at all time points. Finally, to confirm that indeed these changes in gene expression are related to the appearance of a putative β-cell phenotype, we found that the EBs from the H7.Px4 clones produced C-peptide, the fragment cleaved from the Proinsulin peptide during its processing in β-cells (Figure 3D).

### HESC-Derived Cells are Enriched in *Ins* and *Pdx1* Transcripts and Showed Regulated C-Peptide Release to Tolbutamide

**Figure 4:**
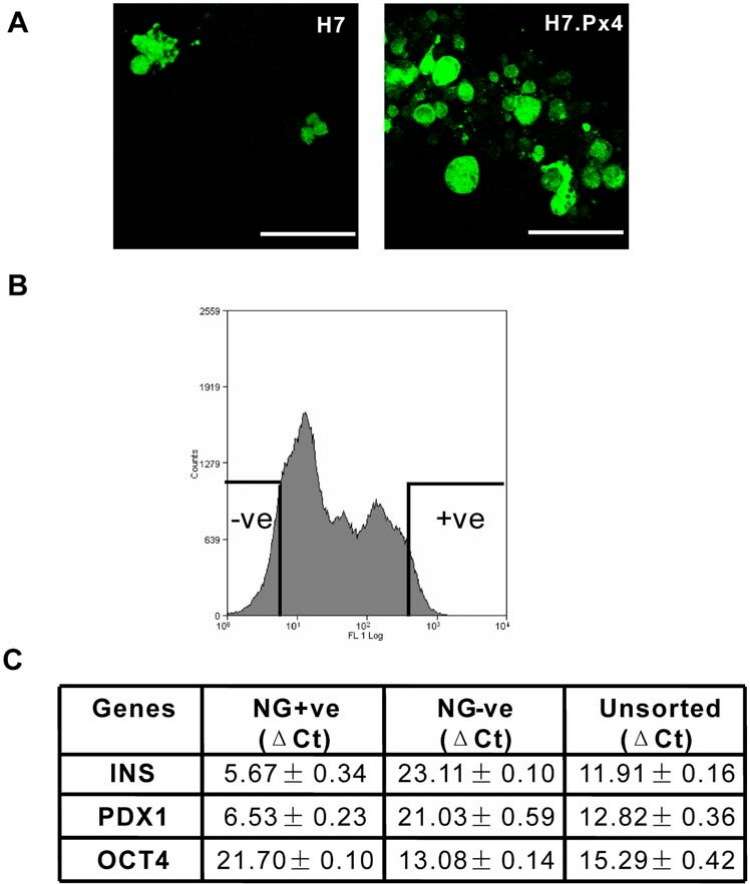
Isolation of putative β-cells based on high zinc content using a vital dye, Newport Green (NG). (A) Confocal microscopic examination of NG staining in H7 and H7.Px4 EB-derived cells. Note the quasi-homogeneous fluorescence of NG-labeled zinc-positive cells with cytoplasmic dots. (B) Isolation of NG+ve (top 10%) and NG-ve (bottom 10%) H7.Px4 cells by FACS. The selected cells were collected and analyzed by Q-PCR for the expression of *Ins*, *Pdx1* and *Oct4*. (C) Gene expression levels in the sorted NG+ve, NG-ve and the unsorted cells are represented as ΔCt values (± standard deviation) relative to *ACTB* for all samples. Note that an increase in ΔCt of 1.0 represents 2-fold decrease in mRNA level. The ΔCt value was determined by subtracting the average housekeeping (*ACTB*) Ct value from the average target genes (*Pdx1*, *Ins* and *Oct4*) Ct values. Error bars represent the standard deviation of the difference, as calculated from the standard deviation of the target and *ACTB* values.

Although the over-expression of Pax4 appears to enhance the formation of cells within the EBs that express features of β-cells, these co-exist with other differentiated derivatives of the HESC, as indicated by the expression of additional genes unrelated to the β-cell lineage (see Supplementary Information, Figure S4). We therefore used fluorescence activated cell sorting (FACS) to isolate the putative β-cells from the H7.Px4 EBs, based upon their distinctive high content of zinc, characteristic of β-cells, which can be detected by staining with the fluorescent vital dye, Newport Green [36]. The EBs were allowed to develop in suspension for 16–21 days, and then plated on Matrigel-coated tissue culture dishes, in low-glucose medium supplemented with nicotinamide for an additional 7–10 days. These cells could then be easily dispersed with trypsin:EDTA and stained with Newport Green, which revealed a heterogeneous intensity of fluorescence with cytoplasmic staining (Figure 4A). Significantly more Newport Green-positive cells were detected by FACS in EBs from the H7.Px4 HESC than from the untransfected H7 cells. When isolated (Figure 4B) and analyzed by Q-PCR these cells were enriched in *Ins* and *Pdx*1 mRNA expression and depleted in expression of *Oct4* relative to presorted and Newport Green-negative population (Figure 4C).

**Figure 5:**
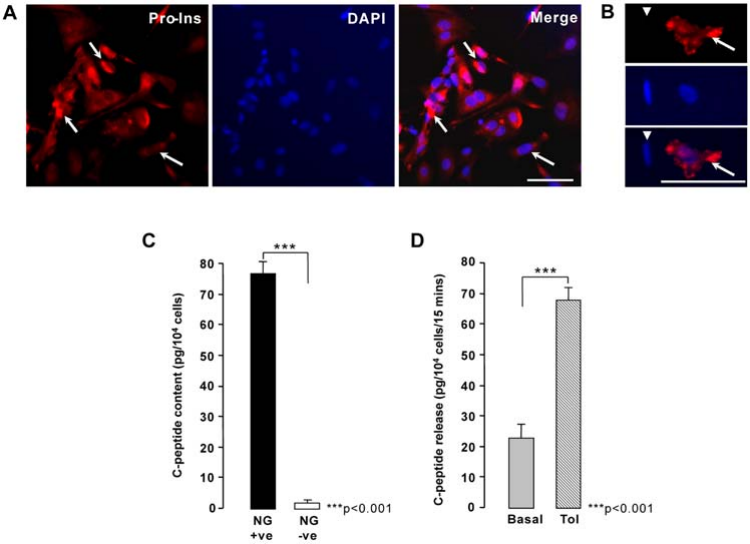
Insulin and C-peptide expression in FACS sorted H7.Px4 cells. H7.Px4 cells sorted for Newport green staining (Figure 4) were replated on human placental collagen VI-treated chambers and cultured for 5 days before immunocytochemistry and C-peptide analysis. (A) shows immunocytochemistry for anti-human Proinsulin antibody (red) with nuclei counterstained with DAPI. Proinsulin-positive reaction is seen scattered in cytoplasm shown in (A) and in a higher magnification in (B) (arrows). Also shown is a Proinsulin-negative cell (arrowhead). Scale bars, 50 µm. (C) and (D) summarize C-peptide content and release in response to 100 µM tolbutamide; the fraction released was 79.92±10.8% from three independent experiments using two H7.Px4 clones. Triple asterisk indicates p<0.001.

After sorting, the Newport Green-positive cells were further cultivated on collagen-IV-coated plates for an additional 5–7 days. More than 90% of these cells could be stained with an antibody to proinsulin, indicating *de novo* insulin synthesis (Figure 5A and 5B) and contained significantly more C-peptide than Newport Green-negative cells (Figure 5C). The difference in C-peptide content and the levels of *Ins* transcripts between the Newport Green-positive and negative cells (36-fold difference) is consistent with the putative β-cells producing equimolar concentrations of insulin and C-peptide [37]. When stimulated with 27.7 mM glucose Newport Green-positive cells failed to respond with C-peptide release (data not shown), but were responsive to tolbutamide, an insulin secretagogue that acts as a K_ATP_-channel inhibitor [38]. In this case, exposure to tolbutamide resulted in C-peptide release by approximately two- to three-fold over non-stimulatory basal levels in three separate experiments (Figure 5D).

## Discussion

The mechanisms that drive the differentiation of HESC when grown in suspension as EBs still remain largely unknown, but typically under these conditions the cells spontaneously generate derivatives of all three primary germ layers [28]. These include a proportion of definitive endoderm and endocrine cells as evidenced by the expression of *Foxa2*, *NeuroD1* and *Isl1* as described in our study. The appearance of low levels of *Ins* transcripts also suggests that these endoderm derivatives may include a few cells resembling β-cells. Previous studies have identified growth factors that promote differentiation of HESC into insulin-producing cells, but few have identified transcription factors that enhance this process in HESC [12]. However, Pax4 has been shown to play a role in pancreatic endocrine and β-cell specification from the early definitive endoderm in mouse embryos and to enhance β-cell differentiation from mouse ES cells [14], [15], [16]. Our data now show that Pax4 also strongly enhances the appearance of putative β-cells in the EBs produced from HESC.

We found no evidence that constitutive expression of Pax4 affects the undifferentiated state of HESC. It is known that HESC do express low baseline levels of lineage-specific markers [28] so when differentiation is triggered by EB formation, the presence of Pax4 or other lineage-specific markers may distort the process of initial lineage selection. However, it seems more likely that the effect of Pax4 expression on β-cell production is due to action later in the differentiation process. The specification of β-cell fate during embryonic development *in vivo* relies on a tightly balanced process of four sequential steps: (1) pancreas precursor specification and proliferation from a definitive endoderm cell pool, (2) pancreas endocrine lineage commitment, (3) formation and differentiation of β-cells, and (4) further maturation into functional glucose-responsive β-cells. We envisaged that the introduction of constitutively expressed Pax4 might bestow a selective advantage on the definitive endoderm cells that form spontaneously in EBs derived from cells and enhance their differentiation towards pancreatic β-cell lineage. For example, since Pax4 over-expression has recently been shown to enhance cell survival [39], constitutive expression of Pax4 may enhance immature endocrine cell survival in addition to promoting endocrine lineage differentiation from endodermal precursors. By this hypothesis, the starting point of Pax4 activation for β-cell differentiation is downstream of endodermal pancreatic induction itself, so that Pax4 activation affects pancreatic endocrine cells that spontaneously differentiated from endoderm in EBs, while other ‘lineage-precommitted’ stem cells are not responsive. Further enhancement of β-cell differentiation may therefore be achieved by regulation of signals that promote or inhibit the initial differentiation of definitive endoderm specification from hES cells, and subsequent specification of the pancreatic lineages. For example, D'Amour et al (2006) have reported that the earliest stages of definitive endoderm differentiation can be modulated by activin A, although Mfopou et al (2007) reported the generation of inhibitory Shh signaling during production of definitive endoderm using activin A [40].

Cells produced in the H7.Px4 EBs exhibited functional properties (C-peptide release and intracellular Ca-signalling responses) typical of human β-cells. We found that Pax4 positively influenced capacity of HESC to respond to depolarizing concentration of KCl in a manner consistent with an action on voltage-gated Ca^2+^ channel gene expression. This was associated with a five fold increase in the proportion of responding cells, such that 14 days after the induction all EBs were functionally responsive to KCl-induced depolarization of the membrane. This was most likely due to the upregulated expression of voltage-dependent Ca^2+^ channel genes including those encoding Cav1.2 and Cav1.3 α-subunits. These genes produce high voltage activated L-type voltage-gated Ca^2+^ channels which are the specific pore-forming subunits of importance in mature pancreatic α- and β-cells and during development [41]. The transcriptional control of VGCC gene expression is not particulary well characterised but it is well known that Ca^2+^ entry via VGCC will influence subsequent gene expression via transcription factors such as CREB, MEF and NFAT which are phosphorylated by Ca^2+^-dependent kinases such as CaMKIV [42]. In addition it has been reported that a C-terminal fragment of Cav1.2 that is produced in developing and adult neurons can regulate the expression of many endogenous genes including ion channels and other proteins of importance for electrically-active cells [42]. Therefore, the early expression of VGCC subunits may be required for normal pancreatic β-cell development and in combination with other signals may trigger the further differentiation of endocrine and β-cell lineages. This hypothesis is supported by studies in the Cav1.3^−/−^ knock-out mouse where loss of pancreatic expression of *CACNA1D* led to low numbers of β-cells in adult mice [43]. Intriguingly, the actions of Pax4 on voltage-gated Ca^2+^ channel function are distinct from the Ca^2+^-signals induced by purinergic receptor agonists,, which appear to be negatively regulated by Pax4 (Figure 3).

In our studies, differentiation of putatitive β-cell progenitors was achieved by outgrowth of late stage EBs on Matrigel and treatment with low glucose and nicotinamide. Similar conditions have previously been demonstrated to enhance human β-cell progenitor differentiation *in vitro* [44]. H7.Px4 EBs produced a higher proportion of Newport Green-positive cells than H7 EBs, and these could be isolated by FACS. Newport Green-positive cells were enriched in *Ins* and *Pdx1* gene expression and had a high content of C-peptide which could be released in a regulated manner, suggesting an increase in the numbers and competency of cells directed towards a β-cell lineage in H7.Px EBs. Most notably, our study demonstrated a strong correlation between the amount of *Ins* transcripts and C-peptide content, which is consistent with the expectation that insulin and C-peptide are produced in equimolar amounts [37]. However, our observations also indicate that Pax4-derived HESC do not respond to glucose stimulation through C-peptide release, but that they are responsive to the insulin secretagogue tolbutamide. Sulphonylurea-mediated responses suggest that these cells have the capacity to express functional ATP-sensitive K-channels and that closure of these channels by tolbutamide facilitates a depolarization of the cell membrane, leading to Ca^2+^-dependent exocytosis of insulin-containing granules. Since glucose failed to replicate this response, it suggests that–as in fetal islets, a key component of the glucose-sensing apparatus of HESC-derived β-cells is functionally absent. The data we present here suggests that this is not at the level of glucose transporter or glucokinase genes, nor the ionic basis of insulin release. In future it will be important to determine whether such cells would develop to a “mature” β-cell phenotype *in situ* following transplantation or whether they will require additional manipulation *in vitro* in order to promote maturation.

In summary, we have described for the first time using HESC how over-expression of Pax4, in combination with simple changes to the cell culture environment, led to the enhanced generation of cells resembling pancreatic β-cells. Further, we were able to purify these cells using FACS and perform functional assays *in vitro* to determine their physiological characteristics. This provides a proof-of-concept that genetic manipulation of HESC can provide a useful tool in the generation of insulin-secreting cells from HESC.

## Supporting Information

Table S1 Oligonucleotide primer sequences used for standard and quantitative PCR in this study. All primers were designed and tested by the authors unless otherwise indicated in Table S1. Primers produced PCR products migrating as single bands of the expected molecular size on agarose gel electrophoresis. PCR products were purified and sequenced to confirm product identity.
(0.09 MB DOC)

Figure S1 Plasmid map of the vector used to transfect HESC. pCAG-PAX4 contains an internal ribosomal entry site (IRES), conferring bicistronic expression of PAX4 coding sequence (CDS; located at 207-1238 base pairs [bp] on Homo sapiens PAX4 mRNA, Gene Bank Accession Number NM_006193) and puromycin-resistance genes, under the control of the CAGG promoter coupled to a PyF101 mutant enhancer.
(0.04 MB DOC)

Figure S2 Differentiation of H7 control and H7.Px4 EBs over a period of 21 days was associated with the expression of Foxa2, a marker of definitive endoderm which persists during pancreatic development. NeuroD1 was expressed earlier in the H7.Px4 EBs relative to controls. Genes encoding subunits of the ATP-sensitive K+ channel (ABCC8 and KCNJ11) which are critical for depolarization-response coupling in β-cells were expressed at all time points in both H7.Px4 and controls. Gcg encoding glucagon gene was upregulated in 2/3 H7.Px4 clones during differentiation but was never detected in H7 control EBs. All data are typical of n = 4 experiments on control H7 cells and EBs, and from n = 1 experiment from each of 3 independent H7.Px4 clones. M, markers; gDNA, genomic DNA; NT, no template control; d0, day 0; +, RT step performed in presence of reverse transcriptase; -, RT step performed in absence of reverse transcriptase.
(0.32 MB DOC)

Figure S3 Somatostatin (SST) transcripts were detected in as early as 7-day differentiation in H7 EBs, however, there were increased levels of SST transcripts in H7.Px4 EBs. This is consistent with the observation that PAX4 commits the early endocrine cells to become somatostatin-producing delta-cells, as in the mouse pancreas (St-Onge et al., 1997).
(0.03 MB DOC)

Figure S4 Spontaneous differentiation of H7.Px4 EBs also gave rise to cells characteristics of all three germ cell layers. RT-PCR analysis of gene expression in EBs produced from untransfected H7 cells and from two independent H7.Px4 clones after 16-21-day in vitro differentiation. Genes characteristic of other lineages such as endoderm (AFP), mesoderm (Coll2) and ectoderm (MAP2) continued to appear in H7.Px4 EBs but was similar to those in untransfected H7 EBs. M, markers.
(0.08 MB DOC)
