# Supplementary material for: PAX4 Enhances Beta-Cell Differentiation of Human Embryonic Stem Cells
Source: PLoS One. 2008 Mar 12;3(3):e1783. doi: 10.1371/journal.pone.0001783 (PMC2262135; doi:10.1371/journal.pone.0001783)
Supplement: Table S1 — Oligonucleotide primer sequences used for standard and quantitative PCR in this study. All primers were designed and tested by the authors unless otherwise indicated in Table S1. Primers produced PCR products migrating as single bands of the expected molecular size on agarose gel electrophoresis. PCR products were purified and sequenced to confirm product identity. (0.09 MB DOC) [file pone.0001783.s001.doc]

**Supplementary Information**

**Table 1. RT-PCR and Q-PCR oligonucleotide primers**

| **Human mRNA** | **Accession Number** | **Primer Sequence** | **Product Size (bp)** | **T_A_ (^o^C)** | **No.**  **of cycles** |
| --- | --- | --- | --- | --- | --- |
| OCT4a | NM_002701 | For-CGACCATCTGCCGCTTTGAG  Rev-CCCCCTGTCCCCCATTCCTA | 577 | 60 | 23 |
| SOX2 | Z31560 | For-CCCCCGGCGGCAATAGCA  Rev-TCGGCGCCGGGGAGATACAT | 448 | 60 | 23 |
|  |  |  |  |  |  |
| PAX4  CDS | NM_006193 | For-ATGAACCAGCTTGGGGGGCTCT  Rev-CTCCTTCCCACTCCCTGCCTCA | 1031 | 52 | 30 |
| PAX4  UTR | NM_006193 | For-CCCGTGAGCCAGCTCTCAAA  Rev-ATGGGCAGGACGGTAAGGACAATG | 569 | 58 | 35 |
| PDX1 | NM_000209 | For-CCCATGGATGAAGTCTACC  Rev-GTCCTCCTCCTTTTTCCAC | 262 | 52 | 35 |
| PDX1  Q-PCR | NM_000209 | For–AGTGATACTGGATTGGCGTTG  Rev–TAGGGAGCCTTCCAATGTGT | 139 | 60 | 40 |
| ISL1 | NM_002202 | For-GGCCAATATTTCCCACTTAGC  Rev-GGCCCTCTCCACCACATC | 520 | 55 | 35 |
| FOXA2 | NM_153675 | For-CCGTTCTCCATCAACAACCT  Rev-GACCCCCACTTGCTCTCTC | 325 | 62.6 | 40 |
| NEUROD1 | NM_002500 | For-CTCGGACTTTTCTGCCTGAG  Rev-GAAGTTGCCATTGATGCTGA | 275 | 62.8 | 30 |
|  |  |  |  |  |  |
| INS | NM_000207 | For-GCCTTTGTGAACCAACACCTG  Rev-GTTGCAGTAGTTCTCCAGCTG | 261 | 58 | 35 |
| INS  Q-PCR | NM_000207 | For–ACCAGCATCTGCTCCCTCTA  Rev– GGTTCAAGGGCTTTATTCCA | 115 | 60 | 40 |
| GCG | NM_002054 | For-TTCTGAGGCCACATTGCTTT  Rev-CTGTGGCTACCAGTTCTTCTATTCT | 290 | 60.2 | 30 |
| KCNJ11 | NM_000525 | For-GAGGACCACAGCCTACTGGA  Rev-TGTAACACCCTGGATGAGCAG | 255 | 60.2 | 30 |
| ABCC8 | NM_000352 | For-TCCTCCAAAAGGTGGTGATG  Rev-TTGTCTGCACGGACGAAG | 190 | 60.2 | 30 |
|  |  |  |  |  |  |
| CACNA1A  Q-PCR | NM_023035 | For-TGCCTGATGATGACAAGACC  Rev-TCGCCAAGATGCCCGTTAG | 183 | 60 | 40 |
| CACNA1B | NM_000718 | For-GCGGAGTTGAGGAAGGAGA  Rev-CATTGCTGAGGGAGGTGGA | 313 | 59 | 32 |
| CACNA1C  Q-PCR | NM_000719 | For-CCTGCTCTTCATCCTGCTCA  Rev-AGGCTCCGTAGGTCGTCATC | 155 | 59 | 40 |
| CACNA1D | NM_000720 | For-CACCTCAACAAATGCCAATCTC  Rev-CATAATAGCGGGTTCTTTTCACAC | 170 | 60 | 32 |
| CACNA1D  Q-PCR | NM_000720 | For-GAAGCGGACCCAACTGAAAG  Rev-CAATCGCATCACTCGGAAAA | 117 | 60 | 40 |
| CACNA1E | NM_000721 | For-ACTCCTCCTTGCGGCTGT  Rev-AACCTGTGCCCTGTCTGTTG | 246 | 59 | 32 |
| CACNA1F | AF067227 | For-GGAGGGAGTGGAGGAGGAAG  Rev-TCTGTGAGGTGGCAAAAGGA | 388 | 60 | 32 |
| CACNA1S | NM_000069 | For-GATGAGGTGACAGTGGGGAAG  Rev-CTGGAGGGGTCTCTGATTGG | 342 | 60 | 32 |
|  |  |  |  |  |  |
| CACNB1 | NM_000723 | For-TCCAAACACCTCAATGTCCA  Rev-TTCCAAGTACTCCGCCAGAT | 128 | 58 | 32 |
| CACNB2 | NM_000724 | For-AAGGGCTACGAGGTCACAGA  Rev-GCGTGCTTACTGGGATTGTT | 146 | 58 | 32 |
| CACNB3 | NM_000725 | For-GCCTCCTTGCCTTTCTCATT  Rev-TCCTCTGTGCCCTGTCTCTC | 156 | 58 | 32 |
| CACNB4 | NM_001005747 | For-CACAGGTTTGATGGGAGGAT  Rev-GTTGTGCTGGGTGATTGATG | 222 | 58 | 32 |
|  |  |  |  |  |  |
| SLC2A1 | NM_006516 | For-CATGTGCTTCCAGTATGTGG  Rev-GTCAGGTTTGGAAGTCTCAT | 312 | 52 | 25 |
| SLC2A2 | L09683 | For-GCAGCTGCTCAACTAATCAC  Rev-TCAGCAGCACAAGTCCCACT | 256 | 60 | 38 |
| GCK | NM_000162 | For-CTGTGACTGAACCTCAAACCCC  Rev-AAGGAGAAGGTGAAGCCCAGG | 621 | 58 | 38 |
| PC1/3 | NM_000439.3 | For- GAGCGAAGAGCCTGGAGTCT  Rev- TGACCCAAAAGGTCATAGCCCAG | 175 | 58 | 33 |
| SST | NM_001048 | For- CTGTGTCACCGGCGCTCCCTC  Rev- GTCTCGCTGAAGACTTGGAGGATTAGG | 383 | 55 | 31 |
|  |  |  |  |  |  |
| COLL2 | NM_001844 | For-GTGTCAGGGCCAGGATGT  Rev-CATCAAATCCTCCAGCCATC | 502 | 60 | 30 |
| MAP2 | NM_031846 | For-AAGAAAGGAGGCCATAAATCAA  Rev-TGCAGCTCCAACTCCTTCAA | 430 | 52 | 35 |
| AFP | NM_001134 | For-ACCTGGCTACCATATTTTTTGCCCAG  Rev-CTCTTAATTCTTTTGTAACTGTTG | 420 | 60 | 32 |
|  |  |  |  |  |  |
| ACTB | NM_001101 | For-ATCTGGCACCACACCTTCTACAATGAGCTGCG  Rev-CGTCATACTCCTGCTTGCTGATCCACATCTGC | 838 | 60 | 25 |
| ACTB | NM_001101 | For-CATCCCCCAAAGTTCACAAT  Rev-CACGAAGGCTCATCATTCAA | 276 | 59.2 | 30 |
| 18sRNA | Ref 1 | For-GTAACCCGTTGAACCCCATT  Rev-CCATCCAATCGGTAGTAGCG | 131 | 59 | 40 |

**Oligonucleotide primer sequences used for standard and quantitative PCR in this study.** All primers were designed and tested by the authors unless otherwise indicated in Table S1. Primers produced PCR products migrating as single bands of the expected molecular size on agarose gel electrophoresis. PCR products were purified and sequenced to confirm product identity.

^1^Wang, R; Li, J; Lyte, K; Yashpal, NK; Fellows, F and Goodyer, CG. Role for β1 integrin and its associated 3, 5, and 6 subunits in development of the human fetal pancreas. *Diabetes* **54**: 2080-2089 (2005).
