# Supplementary material for: PAX4 Enhances Beta-Cell Differentiation of Human Embryonic Stem Cells
Source: PLoS One. 2008 Mar 12;3(3):e1783. doi: 10.1371/journal.pone.0001783 (PMC2262135; doi:10.1371/journal.pone.0001783)
Supplement: Figure S1 — Plasmid map of the vector used to transfect HESC. pCAG-PAX4 contains an internal ribosomal entry site (IRES), conferring bicistronic expression of PAX4 coding sequence (CDS; located at 207-1238 base pairs [bp] on Homo sapiens PAX4 mRNA, Gene Bank Accession Number NM_006193) and puromycin-resistance genes, under the control of the CAGG promoter coupled to a PyF101 mutant enhancer. (0.04 MB DOC) [file pone.0001783.s002.doc]

**Supplementary Information**

**Figure S1** Plasmid map of the vector used to transfect HESC. pCAG-PAX4 contains an internal ribosomal entry site (IRES), conferring bicistronic expression of PAX4 coding sequence (CDS; located at 207-1238 base pairs [bp] on *Homo sapiens* PAX4 mRNA, Gene Bank Accession Number NM_006193) and puromycin-resistance genes, under the control of the CAGG promoter coupled to a PyF101 mutant enhancer.
