# Supplementary material for: PAX4 Enhances Beta-Cell Differentiation of Human Embryonic Stem Cells
Source: PLoS One. 2008 Mar 12;3(3):e1783. doi: 10.1371/journal.pone.0001783 (PMC2262135; doi:10.1371/journal.pone.0001783)
Supplement: Figure S2 — Differentiation of H7 control and H7.Px4 EBs over a period of 21 days was associated with the expression of Foxa2, a marker of definitive endoderm which persists during pancreatic development. NeuroD1 was expressed earlier in the H7.Px4 EBs relative to controls. Genes encoding subunits of the ATP-sensitive K+ channel (ABCC8 and KCNJ11) which are critical for depolarization-response coupling in β-cells were expressed at all time points in both H7.Px4 and controls. Gcg encoding glucagon gene was upregulated in 2/3 H7.Px4 clones during differentiation but was never detected in H7 control EBs. All data are typical of n = 4 experiments on control H7 cells and EBs, and from n = 1 experiment from each of 3 independent H7.Px4 clones. M, markers; gDNA, genomic DNA; NT, no template control; d0, day 0; +, RT step performed in presence of reverse transcriptase; -, RT step performed in absence of reverse transcriptase. (0.32 MB DOC) [file pone.0001783.s003.doc]

**Supplementary Information**

**Figure S2** Differentiation of H7 control and H7.Px4 EBs over a period of 21 days was associated with the expression of *Foxa2*, a marker of definitive endoderm which persists during pancreatic development. *NeuroD1* was expressed earlier in the H7.Px4 EBs relative to controls. Genes encoding subunits of the ATP-sensitive K^+^ channel (*ABCC8* and *KCNJ11*) which are critical for depolarization-response coupling in β-cells were expressed at all time points in both H7.Px4 and controls. *Gcg* encoding glucagon gene was upregulated in 2/3 H7.Px4 clones during differentiation but was never detected in H7 control EBs. All data are typical of n=4 experiments on control H7 cells and EBs, and from n=1 experiment from each of 3 independent H7.Px4 clones. M, markers; gDNA, genomic DNA; NT, no template control; d0, day 0; +, RT step performed in presence of reverse transcriptase; -, RT step performed in absence of reverse transcriptase.
