# Supplementary material for: PAX4 Enhances Beta-Cell Differentiation of Human Embryonic Stem Cells
Source: PLoS One. 2008 Mar 12;3(3):e1783. doi: 10.1371/journal.pone.0001783 (PMC2262135; doi:10.1371/journal.pone.0001783)
Supplement: Figure S3 — Somatostatin (SST) transcripts were detected in as early as 7-day differentiation in H7 EBs, however, there were increased levels of SST transcripts in H7.Px4 EBs. This is consistent with the observation that PAX4 commits the early endocrine cells to become somatostatin-producing delta-cells, as in the mouse pancreas (St-Onge et al., 1997). (0.03 MB DOC) [file pone.0001783.s004.doc]

**Figure S3** Somatostatin (*SST*) transcripts were detected in as early as 7-day differentiation in H7 EBs, however, there were increased levels of *SST* transcripts in H7.Px4 EBs. This is consistent with the observation that PAX4 commits the early endocrine cells to become somatostatin-producing delta-cells, as in the mouse pancreas (St-Onge et al., 1997).
