# Supplementary material for: PAX4 Enhances Beta-Cell Differentiation of Human Embryonic Stem Cells
Source: PLoS One. 2008 Mar 12;3(3):e1783. doi: 10.1371/journal.pone.0001783 (PMC2262135; doi:10.1371/journal.pone.0001783)
Supplement: Figure S4 — Spontaneous differentiation of H7.Px4 EBs also gave rise to cells characteristics of all three germ cell layers. RT-PCR analysis of gene expression in EBs produced from untransfected H7 cells and from two independent H7.Px4 clones after 16-21-day in vitro differentiation. Genes characteristic of other lineages such as endoderm (AFP), mesoderm (Coll2) and ectoderm (MAP2) continued to appear in H7.Px4 EBs but was similar to those in untransfected H7 EBs. M, markers. (0.08 MB DOC) [file pone.0001783.s005.doc]

**Figure S4** Spontaneous differentiation of H7.Px4 EBs also gave rise to cells characteristics of all three germ cell layers. RT-PCR analysis of gene expression in EBs produced from untransfected H7 cells and from two independent H7.Px4 clones after 16-21-day *in vitro* differentiation. Genes characteristic of other lineages such as endoderm (*AFP*), mesoderm (*Coll2*) and ectoderm (*MAP2*) continued to appear in H7.Px4 EBs but was similar to those in untransfected H7 EBs**.** M, markers.
